# Factors influencing recovery of upper limb motor function during constraint-induced movement therapy for people with stroke

**DOI:** 10.1515/tnsci-2022-0260

**Published:** 2022-12-08

**Authors:** Auwal Abdullahi, Bishir Sabo, Umaru Muhammad Badaru, Wim Saeys, Steven Truijen

**Affiliations:** Department of Physiotherapy, Bayero University Kano, Kano, Nigeria; Department of Physiotherapy, Ahmadu Bello University Teaching Hospital, Zaria, Nigeria; Department of Rehabilitation Sciences and Physiotherapy, University of Antwerp, Movant, Wilrijk, Belgium

**Keywords:** stroke, activities of daily living, quality of life, motor recovery, neuropathic pain

## Abstract

**Objective:**

The aim of this study is to determine the personal and clinical factors that can predict recovery of motor function in people with stroke.

**Methods:**

Characteristics of the study participants such as age, sex, time since stroke and type of stroke, motor function, shoulder pain, amount and quality of use of the affected limb in the real world, wrist and elbow spasticity, handedness, central post-stroke pain and dose of massed practice were recorded. The data obtained were analyzed using descriptive statistics and multiple regression.

**Results:**

A total of 144 patients with stroke with mean age, 58.71 ± 19.90 years participated in the study. The result showed that, the whole model significantly explained the total variance by 88.4%, *F*(14, 144) = 32.870, *R* ^2^ = 0. 0.781, *p* < 0.001. However, in the final model, only four independent variables in the order of degree of predictability, amount of use of the limb in the real world (Beta = 0.455, *p* = 0.003), intensity of practice during rehabilitation session (Beta = 0.321, *p* < 0.001), wrist spasticity (Beta = 0.148, *p* = 0.004) and side affected (Beta = 0.093, *p* = 0.033) significantly predicted recovery of motor function.

**Conclusion:**

Encouraging the use of the limb in the real world may be more important than practice during rehabilitation session in the clinic or in the laboratory.

## 1 Introduction

Stroke is a significant cause of limitation or disability in carrying out everyday activities. This is because stroke impairs the functions of the brain such as motor, sensory and cognitive functions [1]. Impairment in motor function can result in limitations in functional ability, problems with balance, increased risk of fall and reduced social participation and quality of life [2–6]. Thus, effective rehabilitation for impairment in motor function following stroke is essential to help the patients overcome these challenges.

To improve the control of movement following stroke, various rehabilitation strategies are used. These include the constraint-induced movement therapy (CIMT) [7,8]. The CIMT is a multi-component rehabilitation intervention; its components include tasks practice, constraint and transfer package [7–9]. The tasks practice is the performance of tasks with the affected the limb, especially the ones that resemble the tasks we carry out in our daily activities [7,9]. The tasks to be performed are usually designed by the therapist or in consultation with the patients [7,8]. The constraint is a technique for constraining the unaffected limb with things such as mitt or sling so that the limb will be limited in use to maximize the chances of using the affected limb [9]. The transfer package is a number of strategies used to maximize the use of the affected limb in everyday activities outside the clinic or the laboratory [8,9].

The CIMT is traditionally administered majorly in the clinic or laboratory and to some extent at home or a combination of clinic or laboratory and home [7,8]. It is reported to improve many outcomes such as motor function, quality of life, balance, persons reported outcomes of health status, real world arm use and ability to perform activities of daily living (ADL) [8,10,11]. However, improvement in motor function may be affected by many factors such as the clinical and personal factors of the patients, like the age, sex, the tasks intensity and the amount and the quality of use of the affected limb in the real world. The aim of this study was to determine which of the following independent variables: sex, age, time since stroke, type of stroke, hand dominance, shoulder pain, wrist spasticity, elbow spasticity, central post-stroke pain (CPSP), side affected, amount of use and quality of use of the affected limb in the real world, intensity of practice and spatial neglect can significantly predict recovery of motor function.

## 2 Methods

### 2.1 Study design, participants and sample size estimation

This was an observational study consisting of patients with stroke in Ahmadu Bello University Teaching Hospital. Details of the inclusion and exclusion criteria used in the study have been published [12].

Adequately trained and qualified physiotherapists at the two sites of Ahmadu Bello University Teaching Hospital carried out the screening of the study participants for eligibility based on the inclusion and exclusion criteria of the study. The physiotherapists who carried out the screening were blinded to the aim of the study. Similarly, adequately trained assessors who were also blinded to the aim of the study carried out outcomes assessment before the participants were asked to carry out tasks practice. The outcomes that were assessed in the study were the demographic and clinical characteristics of the study participants such as age, sex, time since stroke and the types of stroke, motor function, amount and quality of use of the affected limb in the real world, wrist and elbow spasticity, handedness, severity of shoulder pain, intensity of massed practice and central post-stroke pain (CPSP).

Motor function was assessed using Wolf motor function test (WMFT). Amount and quality of use of the limb in the real world was assessed using motor activity log (MAL). Wrist and elbow flexors spasticity was assessed using modified Ashworth scale (MAS). Handedness was assessed using Oldfield handedness questionnaire. CPSP was assessed using Douleur neuropathique 4 questionnaire (DN4Q). Shoulder pain was assessed using visual analogue scale (VAS). The WMFT is a valid and reliable 17 items tool in which the items are scored from zero to five, with the higher scores indicating better motor function ability [13]. The MAL is a valid and reliable tool that consists of two sub-scales that measures the amount and quality of use of the affected limb in the real world [14–17]. Each of the two sub-scales consists of 30 items that are each scored on a scale of zero to five, with the higher scores indicating higher amount and quality of use of the limb in the real world.

The modified MAS is a reliable measure of spasticity that is rated on an ordinal scale of zero to four with the score of zero indicating the absence of spasticity [18]. The DN4Q is a reliable instrument consisting of four items that helps differentiate neuropathic pain from non-neuropathic pain [19,20]. The Oldfield handedness questionnaire is an inventory that consists of 20 items that are rated through direct observation of the individuals’ behavior [21]. The VAS is a 0–10 cm horizontal or vertical line instrument used to assess the severity of patients’ pain from their own perspective [22,23]. The intensity of massed practice was assessed using counting the number of times the task was carried out and the timing with a stop watch.

### 2.2 Data analysis

The characteristics of the study participants were analyzed using descriptive statistics. Relationship between the independent variables was determined using Pearson product moment correlation. Impact (determination of which of the independent variables could significantly predict recovery of motor function) was determined using standard linear multiple regression analysis. The level of significance was set at <0.05. All the analyses were performed using SPSS version 20.


**Ethical approval:** The research related to human use has been complied with all the relevant national regulations, institutional policies and in accordance with the tenets of the Helsinki Declaration, and has been approved by the authors’ institutional review board or equivalent committee (Research Ethics Committee of Ahmadu Bello University Teaching Hospital, Zaria, Nigeria; Approval number, 954524802).
**Informed consent:** Informed consent has been obtained from all individuals included in this study.

## 3 Results

**Figure 1:**
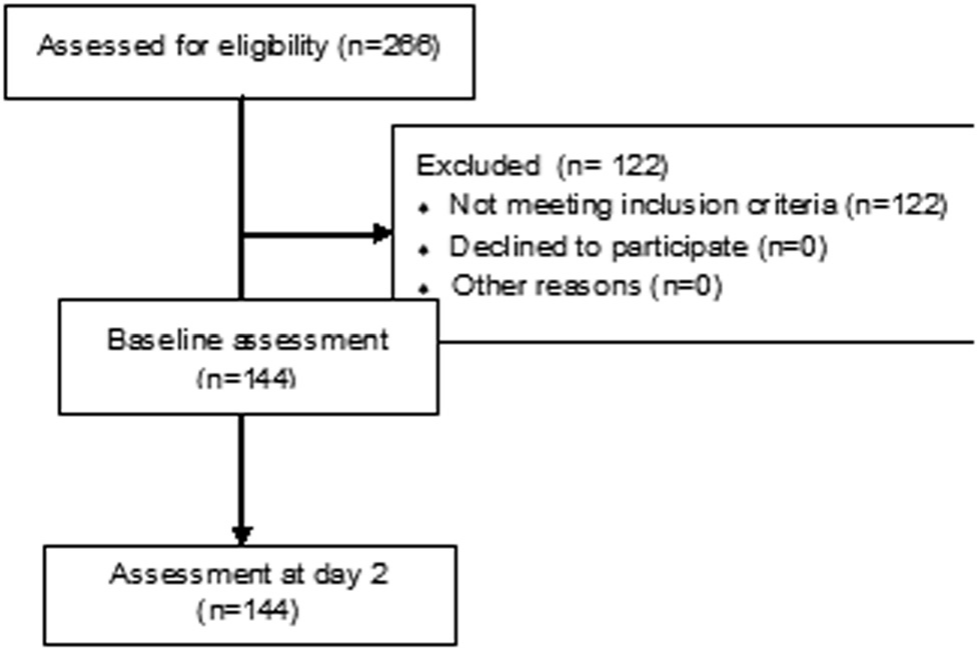
Study flowchart.

A total of 144 patients with stroke with mean age, 58.71 ± 19.90 years participated in the study. The total number of women in the study was 56 (38.9%). See Table 1 for the characteristics of the study participants, and Figure 1 for the study flowchart. In addition, mean value for the observed motor function was 1.96 ± 0.74.

**Table 1:** Characteristics of the study participants

Variable	Mean ± SD	Median (interquartile range)	*n*	%
Sex (M/F)			88/56	61.1/38.9
Type of stroke (I/H)			75/69	52.1/47.9
Dominant hand stroke (R/L)			126/18	87.5/12.5
Side affected (R/L)			101/43	70.1/29.9
Age (years)	58.71 ± 19.90			
Time since stroke (weeks)	36.38 ± 39.99			
Movement quantity	3.00 ± 0.57			
Movement quality	3.05 ± 0.59			
Motor function	1.96 ± 0.74			
Task repetitions	437.50 ± 99.18			
Star cancellation	0.70 ± 0.84			
Star cancellation error		1.00 (1.00)		
Motor function	1.96 ± 0.74			

Key: I/H = ischaemic/haemorrhagic, R/L = right/left.

For the relationship between the independent variables, the result showed that none of the variables was highly correlated with the other (all have *r* < 0.9). Thus, all the variables were entered into the regression model since none of them violated the assumption of multicollinearity. See Table 2 for the details of the relationship between the dependent and independent variables. Similarly, none of the variables was a combination of any two of the independent variables; thus, assumption of singularity was not violated.

**Table 2:** Relationship between the study independent variables (*n* = 144)

Variables	Sex	Age	Time since stroke	Type of stroke	Hand dominance	Shoulder pain	Wrist spasticity	Elbow spasticity	CPSP	Side affected	Amount of use	Quality of use	Intensity of practice	Spatial neglect
Sex														
Age	*r* = 0.063, *p* = 0.452													
Time since stroke	*r* = 0.016, *p* = 0.852	*r* = 0.289, *p* < 0.001												
Type of stroke	*r* = 0.062, *p* = 0.462	*r* = 0.540, *p* < 0.001	*r* = 0.153, *p* = 0.067											
Hand dominance	*r* = 0.00, *p* = 1.00	*r* = −0.006, *p* = 0.941	*r* = 0.021, *p* = 0.806	*r* = −0.110, *p* = 0.188										
Shoulder pain	*r* = −0.023, *p* = 0.875	*r* = 0.157, *p* = 0.006	*r* = 0.045, *p* = 0.589	*r* = 0.116, *p* = 0.168	*r* = −0.040, *p* = 0.634									
Wrist spasticity	*r* = −0.011, *p* = 0.891	*r* = 0.000, *p* = 0.997	*r* = 0.068, *p* = 0.421	*r* = 0.123, *p* = 0.142	*r* = 0.016, *p* = 0.846	*r* = 0.380, *p* < 0.001								

Variables	Sex	Age	Time since stroke	Type of stroke	Hand dominance	Shoulder pain	Wrist spasticity	Elbow spasticity	CPSP	Side affected	Amount of use	Quality of use	Intensity of practice	Spatial neglect
Elbow spasticity	*r* = 0.101, *p* = 0.228	*r* = 0.013, *p* = 0.881	*r* = 0.00, *p* = 0.997	*r* = 0.233, *p* = 0.005	*r* = −0.170, *p* = 0.041	*r* = 0.481, *p* < 0.001	*r* = 0.500, *p* < 0.001							
CPSP	*r* = 0.100, *p* = 0.233	*r* = 0.170, *p* = 0.042	*r* = 0.092, *p* = 0.271	*r* = 0.193, *p* = 0.020	*r* = −0.076, *p* = 0.367	*r* = 0.765, *p* < 0.001	*r* = 0.413, *p* < 0.001	*r* = 0.630, *p* < 0.001						
Side affected	*r* = 0.040, *p* = 0.636	*r* = 0.133, *p* = 0.113	*r* = 0.089, *p* = 0.288	*r* = 0.103, *p* = 0.219	*r* = 0.120, *p* = 0.150	*r* = −0.094, *p* = 0.262	*r* = −0.096, *p* = 0.0253	*r* = −0.152, *p* = 0.069	*r* = −0.155, *p* = 0.064					
Amount of use	*r* = −0.090, *p* = 0.286	*r* = −0.275, *p* = 0.001	*r* = −0.094, *p* = 0.263	*r* = −0.295, *p* < 0.001	*r* = 0.048, *p* = 0.568	*r* = −0.440, *p* < 0.001	*r* = −0.361, *p* < 0.001	*r* = −0.618, *p* < 0.001	*r* = −0.601, *p* < 0.001	*r* = 0.064, *p* = 0.447				
Quality of use	*r* = −0.119, *p* = 0.155	*r* = −0.299, *p* < 0.001	*r* = −0.104, *p* = 0.213	*r* = −0.281, *p* < 0.001	*r* = 0.083, *p* = 0.324	*r* = −0.411, *p* < 0.001	*r* = 0.312, *p* < 0.001	*r* = −0.549, *p* < 0.001	*r* = −0.556, *p* < 0.001	*r* = 0.066, *p* = 0.431	*r* = 0.948, *p* < 0.001			
Intensity of practice	*r* = −0.161, *p* = 0.054	*r* = −0.366, *p* < 0.001	*r* = −0.162, *p* = 0.053	*r* = −0.332, *p* < 0.001	*r* = −0.075, *p* = 0.370	*r* = −0.472, *p* < 0.001	*r* = −0.421, *p* < 0.001	*r* = −0.556, *p* < 0.001	*r* = −0.548, *p* < 0.001	*r* = 0.010, *p* = 0.901	*r* = 0.823, *p* < 0.001	*r* = 0.797, *p* < 0.001		
Spatial neglect	*r* = −0.023, *p* = 0.788	*r* = −0.451, *p* < 0.001	*r* = −0.090, *p* < 0.001	*r* = −0.316, *p* < 0.001	*r* = −0.006, *p* = 0.941	*r* = −0.516, *p* = 0.062	*r* = −0.014, *p* = 0.872	*r* = 0.002, *p* = 0.982	*r* = −0.088, *p* = 0.295	*r* = −0.118, *p* = 0.159	*r* = 0.152, *p* = 0.069	*r* = 0.149, *p* = 0.075	*r* = 0.264, *p* = 0.001	

Key: I/H = ischemic/hemorrhagic, R/L = right/left.

**Figure 2:**
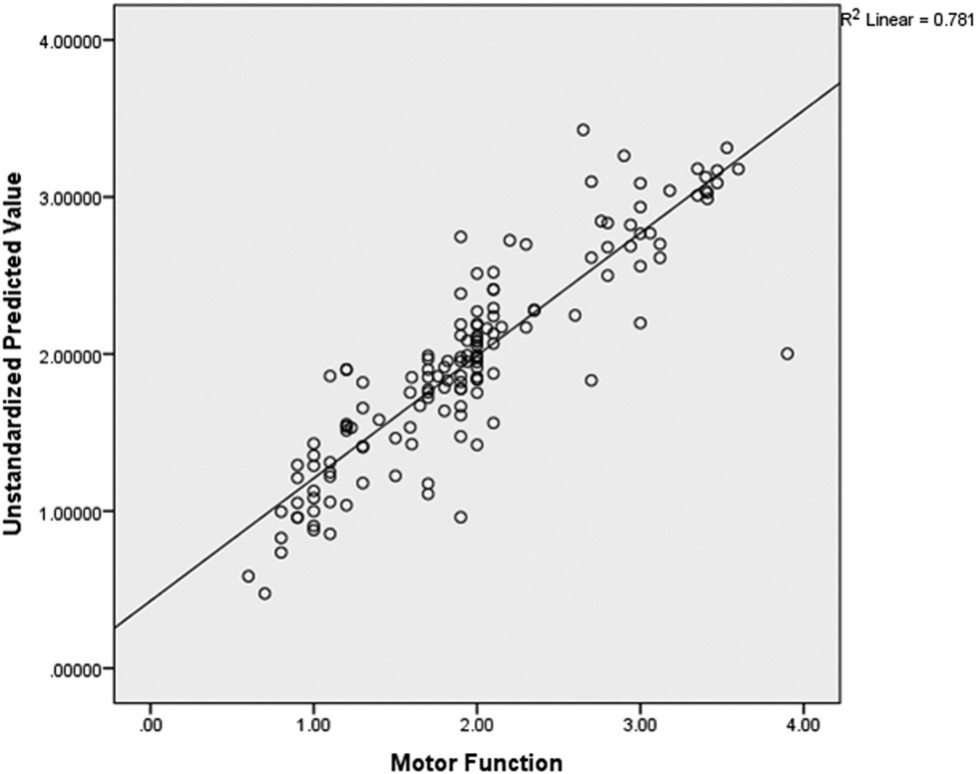
Scatter plot indicating the regression line.

For the standard multiple regression analysis, the total variance explained by the whole model was significant, 88.4%, *F*(14, 144) = 32.870, *R* ^2^ = 0. 0.781, *p* < 0.001, with a predicted mean value of motor function, 1.96 ± 0.66. This shows that, based on the *R* ^2^ and *p*-values obtained, there was a significant and a strong correlation between the observed and predicted mean (1.96 ± 0.74) values of motor function scores. However, in the final model, only four independent variables, i.e., amount of use of the limb in the real world (Beta = 0.455, *p* = 0.003), intensity of practice during rehabilitation session (Beta = 0.321, *p* < 0.001), wrist spasticity (Beta = 0.148, *p* = 0.004) and side affected (Beta = 0.093, *p* = 0.033) significantly predicted the recovery of motor function. See Table 3 and Figure 2 for the details of the results of this analysis.

**Table 3:** Predictors of recovery of motor function following CIMT

Variable	*B*	*r*	95% CI	*p*
Sex	0.044	−0.092	−0.086 to 0.174	0.506
Age	0.001	−0.693	−0.003 to 0.005	0.673
Time since stroke	0.001	−0.059	−0.001 to 0.002	0.524
Type of stroke	−0.121	−0.313	−0.274 to 0.032	0.121
Hand dominance	0.174	−0.031	−0.371 to 0.023	0.084
Shoulder pain	0.025	−0.366	−0.050 to 0.101	0.511
Wrist spasticity	0.227	−0.246	0.075 to 0.380	0.004*
Elbow spasticity	−0.123	−0.555	−0.302 to 0.055	0.173
CPSP	−0.007	−0.514	−0.091 to 0.077	0.868
Side affect	0.150	0.124	0.012 to 0.288	0.033*
Amount of use	0.599	0.850	0.212 to 0.987	0.003*
Quality of use	0.174	0.822	−0.162 to 0.511	0.308
Intensity of practice	0.002	0.787	0.001 to 0.004	<0.001*
Spatial neglect	−0.028	0.130	−0.113 to 0.056	0.509

*Significant at *p* < 0.05.

## 4 Discussion

The aim of this study was to determine the predictors of recovery of motor function following the use of CIMT in people with stroke. The result showed that only the amount of use of the limb in the real world, intensity of practice during rehabilitation session, wrist spasticity and side affected significantly predicted the recovery of motor function. Both the amount of use of the limb in the real world and intensity of practice during rehabilitation session signify how often the limb is used. Repetitive tasks practice with the affected limb after stroke can result in use-dependent plasticity [24]. However, since use of the limb in the real world is the highest predictor of recovery of motor function, the findings seem to suggest that, CIMT protocol should be tailored to the return of routine daily activities as soon as possible. For instance, patients should be encouraged to return to their routine cooking, bathing, washing, eating, walking and writing activities with the affected limb during motor rehabilitation. This is because these activities are done in the real-life situation, and their memory is already stored in the brain. Thus, they may induce biochemical, anatomical and functional changes in the brain better to facilitate the recovery of motor function [25].

In addition, failure to return to work is associated with negative outcomes such as depression, increased risks of cardiac disease, mortality and poor coping ability [26]. Interestingly, all the three main components of CIMT, tasks practice with the affected limb, constraint of the unaffected limb and the transfer package are meant to maximize the use of the affected limb. However, considering the present result, a special emphasis should be given to the transfer package in order to tailor the rehabilitation package to practice in the real-life situation. In addition, the tasks practice should be started early post-stroke to coincide with the natural occurrence of these biomarkers [24].

Another important finding of this study is that wrist spasticity significantly predicted the recovery of motor function. This is not surprising since wrist spasticity can prevent the use of the affected limb [27,28]. When the motor function of distal part of the limb is impaired probably as a result of wrist spasticity, it may be an indicator of the affectation of the distal part of the corticospinal tract [29]. Corticospinal tract is the medium through which nerve impulses generated by the primary motor cortex (M1) are transmitted to the motor units, which are connected to the skeletal muscles [4,30]. Thus, other rehabilitation strategies to improve spasticity should be combined with CIMT post-stroke. This is to prevent wrist spasticity from negatively influencing the recovery of motor function in patients with stroke. Furthermore, lateralization can serve as an indicator of recovery. This is because, those with right hemispheric stroke showed better cortical facilitation in the contralesional hemisphere compared to those with left hemispheric stroke [31,32]. However, recovery may also be related to limb dominance as the dominant limb tends to show higher cortical activity [29]. Although the relatively large sample size of this study is an important strength of the study, lack of a control may undermine the credibility of the findings. Thus, the findings of the study need to be interpreted with caution.

## 5 Conclusion

Encouraging the use of the limb in the real world may be more important than practice during rehabilitation session in the clinic or in the laboratory. This is because people have more time for their daily activities than for rehabilitation in the clinic. Thus, it is important that upper limb rehabilitation is centered on the patients’ ADL. Also distal limb motor function is an indicator of spared function of the distal corticospinal tract. However, this can be impaired in the presence of spasticity. Therefore, improving wrist spasticity is important for recovery.
